# Ambulatory electrocardiographic longitudinal monitoring in a canine model for Duchenne muscular dystrophy identifies decreased very low frequency power as a hallmark of impaired heart rate variability

**DOI:** 10.1038/s41598-024-59196-z

**Published:** 2024-04-18

**Authors:** Inès Barthélémy, Jin Bo Su, Xavier Cauchois, Frédéric Relaix, Bijan Ghaleh, Stéphane Blot

**Affiliations:** 1 “Biology of the Neuromuscular System” Team, U955 IMRB, INSERM, Univ Paris-Est Créteil, 94010 Créteil, France; 2 École Nationale Vétérinaire d’Alfort, IMRB, 7 Avenue du Général de Gaulle, 94700 Maisons-Alfort, France; 3 Inserm U955-IMRB, UPEC, Ecole Nationale Vétérinaire d’Alfort, Créteil, France

**Keywords:** Translational research, Preclinical research, Experimental models of disease, Neuromuscular disease, Electrocardiography - EKG

## Abstract

Duchenne muscular dystrophy (DMD) patients exhibit a late left ventricular systolic dysfunction preceded by an occult phase, during which myocardial fibrosis progresses and some early functional impairments can be detected. These latter include electrocardiographic (ECG) and heart rate variability (HRV) abnormalities. This longitudinal study aimed at describing the sequence of ECG and HRV abnormalities, using Holter ECG in the GRMD (Golden retriever muscular dystrophy) dog model, known to develop a DMD-like disease, including cardiomyopathy. Most of the known ECG abnormalities described in DMD patients were also found in GRMD dogs, including increased heart rate, prolonged QT and shortened PR intervals, ventricular arrhythmias, and several of them could be detected months before the decrease of fractional shortening. The HRV was impaired like in DMD patients, one of the earliest evidenced abnormalities being a decrease in the very low frequency (VLF) component of the power spectrum. This decrease was correlated with the further reduction of fractional shortening. Such decreased VLF probably reflects impaired autonomic function and abnormal vasomotor tone. This study provides new insights into the knowledge of the GRMD dog model and DMD cardiomyopathy and emphasizes the interest to monitor the VLF power in DMD patients, still unexplored in this disease, whilst it is highly predictive of deleterious clinical events in many other pathological conditions.

## Introduction

Duchenne Muscular Dystrophy (DMD) is an X-linked genetic disorder affecting 15.9 to 19.6 boys over 100,000 male births and is due to mutations in the *DMD* gene^1^. In skeletal muscles and heart, dystrophin is localized beneath the sarcolemma and stabilizes the cell membrane during contraction. In the absence of dystrophin, the membrane becomes damaged upon mechanical stress and a cascade of events leads to cell degeneration^2^. Clinically, DMD is characterized by prominent muscle weakness leading to locomotor disabilities, progressing in the childhood. Complete loss of ambulation occurs around 10 years of age without any therapeutic intervention^1^. In the second decade, respiratory muscle weakness progresses, accelerated by the transition to wheelchair, and necessitates respiratory assistance^3^. Thanks to the progresses made in the medical management of DMD patients over the last decades, in particular in ventilatory assistance, survival of patients has increased by a decade, making the DMD cardiomyopathy, that appears later than skeletal muscle disabilities, a growing medical issue and a major therapeutic target^4,5^.

Therefore, standards of care for DMD patients now include a yearly comprehensive cardiac evaluation from time of diagnosis, in order to detect the onset of left ventricular (LV) ejection fraction (LVEF) or fractional shortening (LVFS) decrease, usually in the mid-teens, and adapt the therapeutic management^3^. It is however now well known that before the LVEF decrease, the dystrophin-deficient heart undergoes an “occult” phase during which some early abnormalities can be detected and could help anticipate the drop of LVEF. It is the case of myocardial fibrosis that can be detected months before LV global systolic dysfunction (*i.e*., decrease of the LVEF or LVFS) using cardiac MRI and late gadolinium enhancement (LGE) mapping, and can predict the decrease of LVEF^6,7^. Some other studies have shown that early echocardiographic abnormalities can be evidenced in young DMD patients with a normal LVEF. Tissue doppler imaging (TDI) technique allows the identification of an early decrease of radial strain rate (endo-epicardial gradient of velocity) and peak systolic strain of the LV free wall^8^.

Electrocardiograms (ECG) can also detect abnormalities long before the decrease of LVEF, and yearly ECGs are part of the standards of care for DMD patients^3,9,10^. Among typical ECG abnormalities, PR shortening, prolonged QT, increased heart rate are found, as well as deep Q-waves and tall R waves on some leads^9–11^. Fragmented QRS can also be found, and are associated with the degree of LV dysfunction and myocardial fibrosis^12^. A special attention is paid to the presence of arrhythmic events on DMD patients’ ECGs. These arrhythmias can be of supraventricular or ventricular origin, and premature ventricular beats can lead to ventricular tachycardia (VT) episodes, or even fibrillation, and increase the risk of sudden death^13^. Holter electrocardiograms are recommended to detect such arrhythmic events^3^. During the early occult phase of cardiomyopathy, heart rate variability (HRV) abnormalities can be detected. The studies that focused on this aspect used mostly 24 h Holter ECGs. They showed a decreased HRV and evidenced an autonomic dysfunction, with increased sympathetic and decreased parasympathetic modulation, that occurs before LV global systolic dysfunction, together with myocardial fibrosis, and aggravates with advanced stages of the disease^14–21^. HRV decrease has been associated with many other conditions as a prognostic marker of a poor outcome and mortality^22,23^. It has been suggested to use HRV analysis more widely in DMD patients, to improve the early detection of the cardiac disease. However, the sequential evolution of all the early abnormalities that ultimately lead to impaired heart function and the way they interact together in DMD are not yet fully characterized.

To better understand the human DMD, and assess therapeutic strategies, animal models can be useful to provide relevant information. In the field of DMD cardiomyopathy, the canine GRMD (Golden retriever muscular dystrophy) model is noticeably relevant. Indeed, these dogs develop a cardiomyopathy that resembles the human one in many aspects. First histological lesions and cardiac dysfunction parallel those found in the human DMD, both in terms of nature and of timeline^24–28^. Indeed, LV global systolic dysfunction (*i.e*., significant decrease of the LVEF or LVFS relative to healthy controls and under the low value of the normal range) occurs at a late stage (respectively after 18 and 24 months of age), long after the onset of locomotor and respiratory signs. Secondly, a cardiac dysfunction can also be detected very early, using TDI-echocardiography, and strain rate decrease notably^25,29^. Third, myocardial fibrosis can be detected non-invasively using MRI and LGE^27^, progresses from 6 months of age, and becomes prominent between 12 and 24 months of age^28^. At last, DMD-like ECG abnormalities were described, including deep Q-waves, sinus tachycardia, shortened PR, and premature ventricular contractions^26,30^. However, no ECG longitudinal study was performed on the long-term and using Holter ambulatory recordings, and correlation with cardiac function data are lacking. Furthermore, no HRV assessment was ever performed in GRMD dogs.

This study, based on longitudinal Holter ECG data analysis, including HRV assessment, aimed at describing the sequential events of cardiac involvement in this model to improve its knowledge and optimize its use in preclinical studies, by identifying early indicators of the cardiomyopathy. It also aimed at assessing possible correlations of ECG abnormalities with already available echocardiography data^25^, which could be translationally relevant, to improve the knowledge on the DMD cardiomyopathy.

## Results

### GRMD dogs

Fifteen GRMD dogs were included in this study at the age of 2 months, and underwent iterative Holter ECGs. Four of them were euthanized around six months of age due to a loss of ambulation. Two dogs were euthanized around 9 months of age. A seventh dog was euthanized at the age of 15 months. Three dogs died in their 3^rd^ year. One died at 36 months of age around one hour after uncomplicated recovery from general anesthesia, after a short syncopal episode followed by cardiorespiratory arrest and failed resuscitation attempt. The four last GRMD dogs all survived until the 60 months timepoint. For two of them, decompensated heart failure occurred respectively at 63 and 65 months of age, and manifested as ascites, pleural effusion and pulmonary edema for one dog. A third one had to be euthanized at 70 months of age due to idiopathic pericardial effusion. For the last surviving GRMD dog of this cohort, decompensated heart failure occurred at the age of 78 months. A survival curve is available in the Fig. S1 together with a more detailed description of the causes of death of the animals. The number of GRMD dogs per age and the main results are available in Tables 1 and S2.

**Table 1:** Summary of the main results.

Variable	Group	Age (months)			
		2	6	12	24
	Healthy n =	8	9	6	6
	GRMD n =	15	15	9	8
HR (bpm)	Healthy, mean (± SD)	133.4 (± 26.2)	105.8 (± 8.6)	79.4 (± 4.0)	68.0 (± 4.6)
	GRMD, mean (± SD)	156.6 (± 19.4)	114.4 (± 9.2)	99.5 (± 7.4)	90.1 (± 10.1)
	Fisher LSD p-value	**0.049**	**0.032**	** < 0.001**	** < 0.001**
QTcV (ms)	Healthy, mean (± SD)	238.6 (± 7.7)	232.4 (± 3.2)	230.1 (± 8.3)	226.1 (± 3.6)
	GRMD, mean (± SD)	230.9 (± 12.2)	239.4 (± 7.2)	243.7 (± 8.4)	245.1 (± 7.9)
	Fisher LSD p-value	0.076	**0.004**	**0.010**	** < 0.001**
PR (ms)	Healthy, mean (± SD)	75.4 (± 10.8)	87.7 (± 9.4)	101.5 (± 8.5)	110.9 (± 8.6)
	GRMD, mean (± SD)	71.5 (± 8.4)	82.3 (± 8.2)	95.9 (± 8.0)	98.0 (± 8.6)
	Fisher LSD p-value	0.396	0.175	0.231	**0.018**
pNN50 (%)	Healthy, mean (± SD)	39.3 (± 24.7)	56.9 (± 14.7)	77.1 (± 5.0)	80.1 (± 6.1)
	GRMD, mean (± SD)	20.9 (± 14.7)	57.7 (± 9.0)	62 (± 4.3)	67.5 (± 9.2)
	Fisher LSD p-value	0.082	0.876	** < 0.001**	**0.010**
STV (ms)	Healthy, mean (± SD)	52.3 (± 35.0)	105.6 (± 46.0)	197.8 (± 56.1)	216.7 (± 66.3)
	GRMD, mean (± SD)	25.7 (± 14.8)	96.0 (± 24.6)	126.3 (± 26.4)	148.4 (± 19.7)
	Fisher LSD p-value	0.073	0.575	**0.024**	0.053
STV/LTV	Healthy, mean (± SD)	0.45 (± 0.19)	0.56 (± 0.14)	0.62 (± 0.06)	0.67 (± 0.13)
	GRMD, mean (± SD)	0.33 (± 0.11)	0.53 (± 0.12)	0.45 (± 0.07)	0.42 (± 0.04)
	Fisher LSD p-value	0.144	0.554	** < 0.001**	**0.004**
VLF (ms^2^)	Healthy, mean (± SD)	1156.6 (± 565.8)	4973.7 (± 2369.6)	14,323.7 (± 8969.4)	16,258.1 (± 10,777.8)
	GRMD, mean (± SD)	455.3 (± 246.8)	2007.1 (± 1013.8)	4116.9 (1402.8)	6411.2 (± 1699.3)
	Fisher LSD p-value	**0.009**	**0.005**	**0.038**	0.075
LF (ms^2^)	Healthy, mean (± SD)	1378.5 (± 733.2)	4201.8 (± 2281.2)	18,229.4 (± 14,751.0)	18,720.7 (± 15,239.6)
	GRMD, mean (± SD)	539.3 (± 354.4)	5028.4 (± 6795.7)	27,170.5 (± 23,400.2)	47,926.1 (± 25,972.1)
	Fisher LSD p-value	**0.014**	0.722	0.381	**0.022**
HF (ms^2^)	Healthy, mean (± SD)	5836.2 (± 4811.8)	20,172.9 (± 14,020.1)	44,279.7 (± 28,620.1)	39,144.0 (± 23,290.1)
	GRMD, mean (± SD)	1561.7 (± 1530.0)	17,660.2 (± 6967.4)	28,834.3 (± 12,939.3)	32,883.1 (± 13,091.0)
	Fisher LSD p-value	**0.041**	0.649	0.259	0.572
Total power	Healthy, mean (± SD)	8371.3 (± 5952.5)	29,348.4 (± 18,010.3)	76,832.8 (± 47,887.6)	74,122.8 (± 41,005.4)
	GRMD, mean (± SD)	2556.3 (± 2081.7)	24,745.6 (± 12,481.1)	60,121.7 (± 28,256.8)	87,220.4 (± 18,761.7)
	Fisher LSD p-value	**0.028**	0.511	0.465	0.492
HF n.u. (%)	Healthy, mean (± SD)	67.4 (± 24.1)	80.4 (± 4.8)	72.0 (± 8.7)	68.4 (± 12.0)
	GRMD, mean (± SD)	68.9 (± 11.3)	81.4 (± 10.8)	55.9 (± 22.7)	43.7 (± 22.3)
	Fisher LSD p-value	0.868	0.762	0.080	**0.022**

*HR* heart rate, *QTcV* QT interval—Van de Water’s correction, *pNN50* percentage of interval differences of successive RR intervals of more than 50 ms, *STV* short-term variability (Poincaré plot), *STV/LTV* short-term/long-term variability (Poincaré plot), *VLF* very low frequency power, *LF* low frequency power, *HF* high frequency power, *HF n.u.* HF in normalized units (HF/HF + LF).

Significant values are in [bold].

### ECG analysis

**Figure 1:**
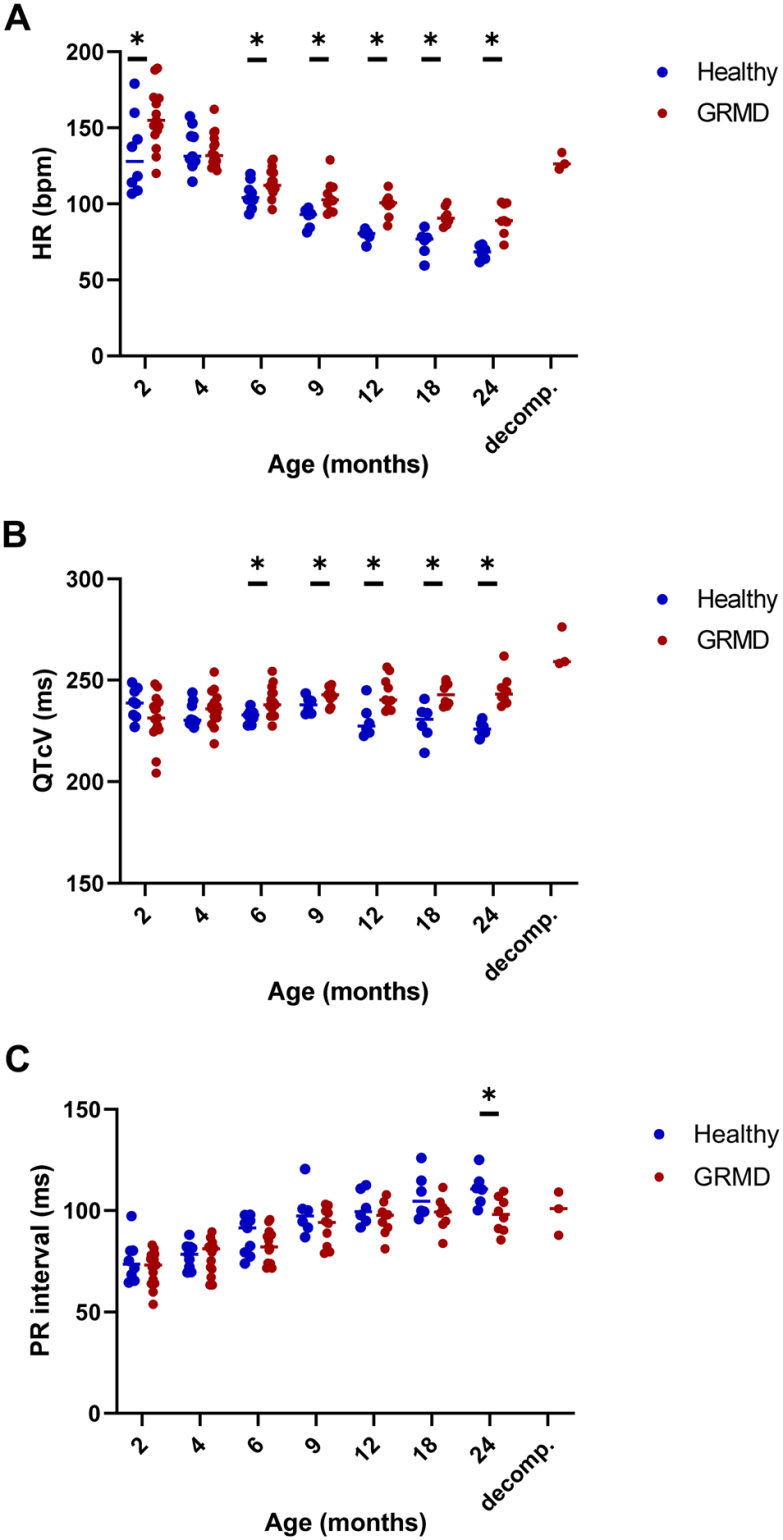
ECG analysis—evolution of intervals and waves ratio with age. On all the graphs, the evolution of GRMD dogs’ values are represented by red dots, and healthy dogs’ values by blue dots. A line indicates the median of each population at each timepoint. Asterisks symbolize a significant difference between GRMD and healthy dogs at a given timepoint (post-hoc Fisher LSD test *p* < 0.05). (**A**) Heart rate (HR) (**B**) QTcV (Van de Water’s correction) interval (**C**) PR interval.

**Heart rate** (HR) significantly decreased with age in both healthy and GRMD dogs and was significantly higher in GRMD dogs than in healthy dogs over the 2–24 months period (*p* < 0.001) (Fig. 1A). GRMD dogs exhibited significantly higher HR relative to healthy littermates at 2 months of age, and from the age of 6 months. From the age of 12 months, the means of the HR in the two groups differed from 19.9 bpm on average (SD = 1.9 bpm). A marked increase in HR was observed in the three dogs with decompensated heart failure.

**The QT interval** significantly increased with age in both groups, consistently with the HR decrease (Fig. S3). No significant difference was found between healthy and GRMD dogs. Given the significant variations in HR with age and between groups, and the known impact of HR on the QT interval, the use of a corrected QT (QTc) appeared necessary to compare both groups. A preliminary study intending to determine the best QT correction formula for our cohort was performed, and based on the HR and four different QTc values obtained in the healthy dogs (Fig. S3). A significant negative correlation between QT and HR was confirmed (R = − 0.929, slope of the regression line -0.59). As also described in dogs, QTcB, the correction formula used in most of the studies in humans, was found to strongly overcorrect QT values (R = 0.881, slope = 0.53)^31^. The QTc with the R and slope values of the regression line closest to 0 was QTcV (R = 0.171, slope of the regression line = 0.04). QTcV was thus selected for the analysis.

A significant increase of QTcV with age was found both in healthy and GRMD dogs and QTcV was significantly increased in GRMD dogs compared to healthy dogs over the 2–24 months period (*p* = 0.002). Post-hoc tests revealed a significant increase of QTcV values in GRMD dogs relative to healthy dogs from the age of 6 months (Fig. 1B). In the three dogs with decompensated heart failure, the QTcV increased by 16.1 ms on average (SD = 6.2 ms) from their values at 60 months of age (Fig. S4).

**The PR interval** was found significantly shorter in GRMD dogs over the 2–24 months period (*p* = 0.035), and significantly increased with age in both groups (*p* < 0.001) (Fig. 1C). Post-hoc analysis revealed that the PR interval was significantly decreased in GRMD dogs relative to healthy dogs only at the latest timepoint (24 months of age) (*p* = 0.018).

**Figure 2:**
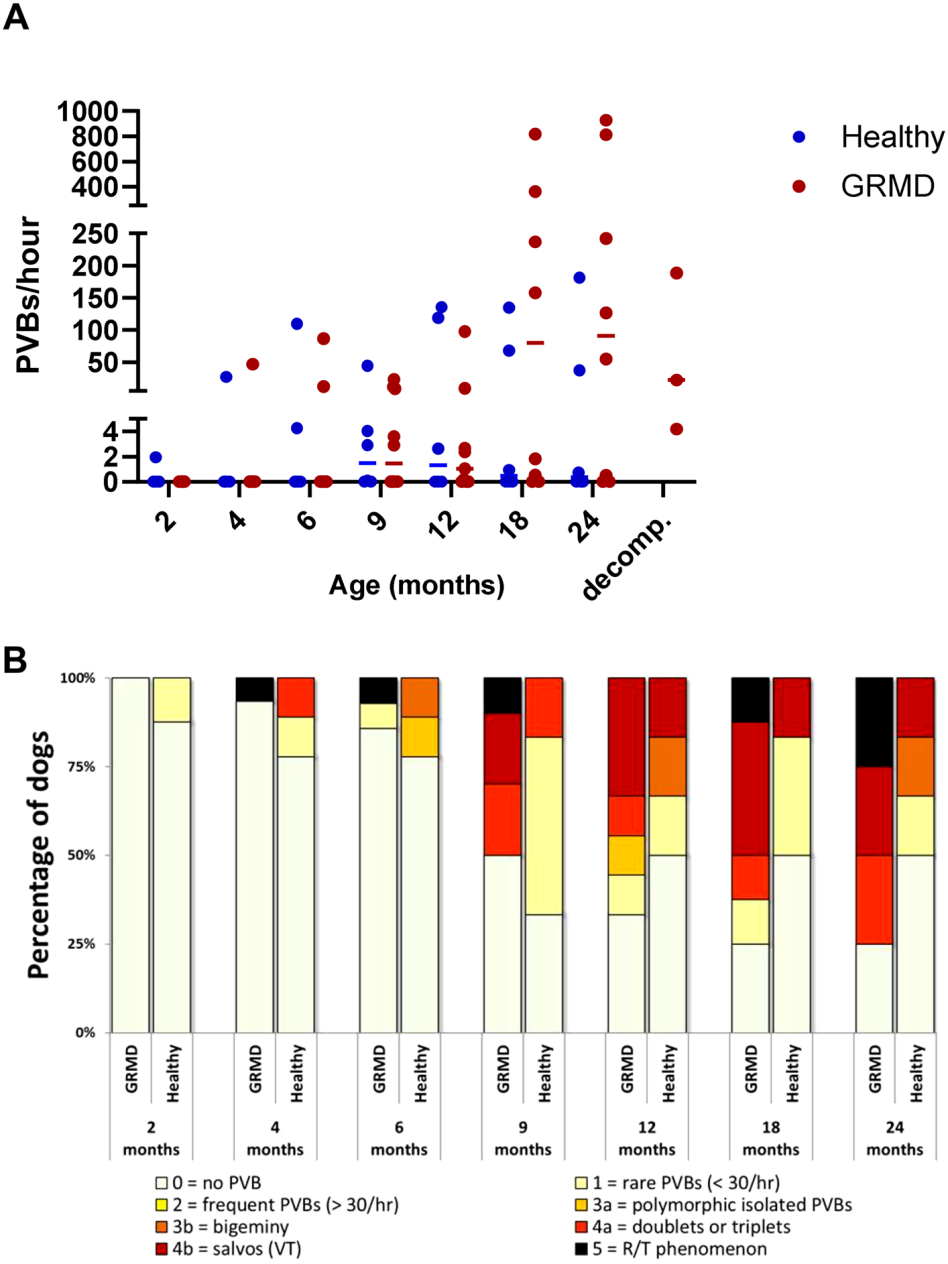
Ventricular arrhythmias in GRMD dogs. (**A**) Evolution of the frequency of premature ventricular beats (PVBs, in percentage of the total beats on the trace) with age in GRMD dogs (red dots), versus healthy dogs (blue dots). Lines indicate the median of the population at each timepoint. (**B**) Lown classification of PVBs severity shows that the proportion of dogs with PVBs tends to increase with age in GRMD dogs. Severity varies among dogs but can reach high levels with some dogs presenting with ventricular tachycardia or R/T PVBs (about 50% GRMD dogs from the age of 18 months).

**Arrhythmic events** were frequently detected in GRMD ECG, and most of them were of ventricular origin, though supraventricular arrhythmias or conduction defects were found sporadically. Premature ventricular beats (PVBs) were a common finding in GRMD dogs, and their frequency (in beats per hour) increased with age (*p* = 0.006) (Fig. 2). PVBs were absent from ECGs of 2-month-old GRMD dogs but were found at all ages afterwards and concerned 50% of the GRMD dogs at the age of 9 months. The severity of these PVBs, according to the Lown classification, increased with age. The two highest grades of the Lown classification, corresponding to ventricular tachycardia salvos, and R/T phenomenon were reached for some GRMD dogs at several timepoints, evidencing a risk of sudden deaths in these animals. In this cohort, it was the case for one dog that suddenly died without any identified cause, few days after the 36 months Holter during which the dog presented some episodes of ventricular tachycardia (VT).

It is noteworthy that in the healthy dogs group, three individuals presented with PVBs, which occurred relatively frequently for two of them and could manifest as doublets, triplets and salvos for one of them, leading to high scores on the Lown scale. However, PVBs seen in these healthy dogs were always monomorphic (and conserved the same morphology across the different timepoints) while GRMD dogs tended to exhibit polymorphic PVBs. The VT salvos seen in one healthy dog were not sustained, comprising usually 4–5 contractions, and a maximum of 10, once.

**Fragmented QRS **were found in one GRMD dog at 9 months of age, and in four GRMD dogs (half the group) from the age of 24 months. The longest survivor had fragmented QRS detected only at time of decompensated heart failure.

### Heart rate variability (HRV) analysis

**Figure 3:**
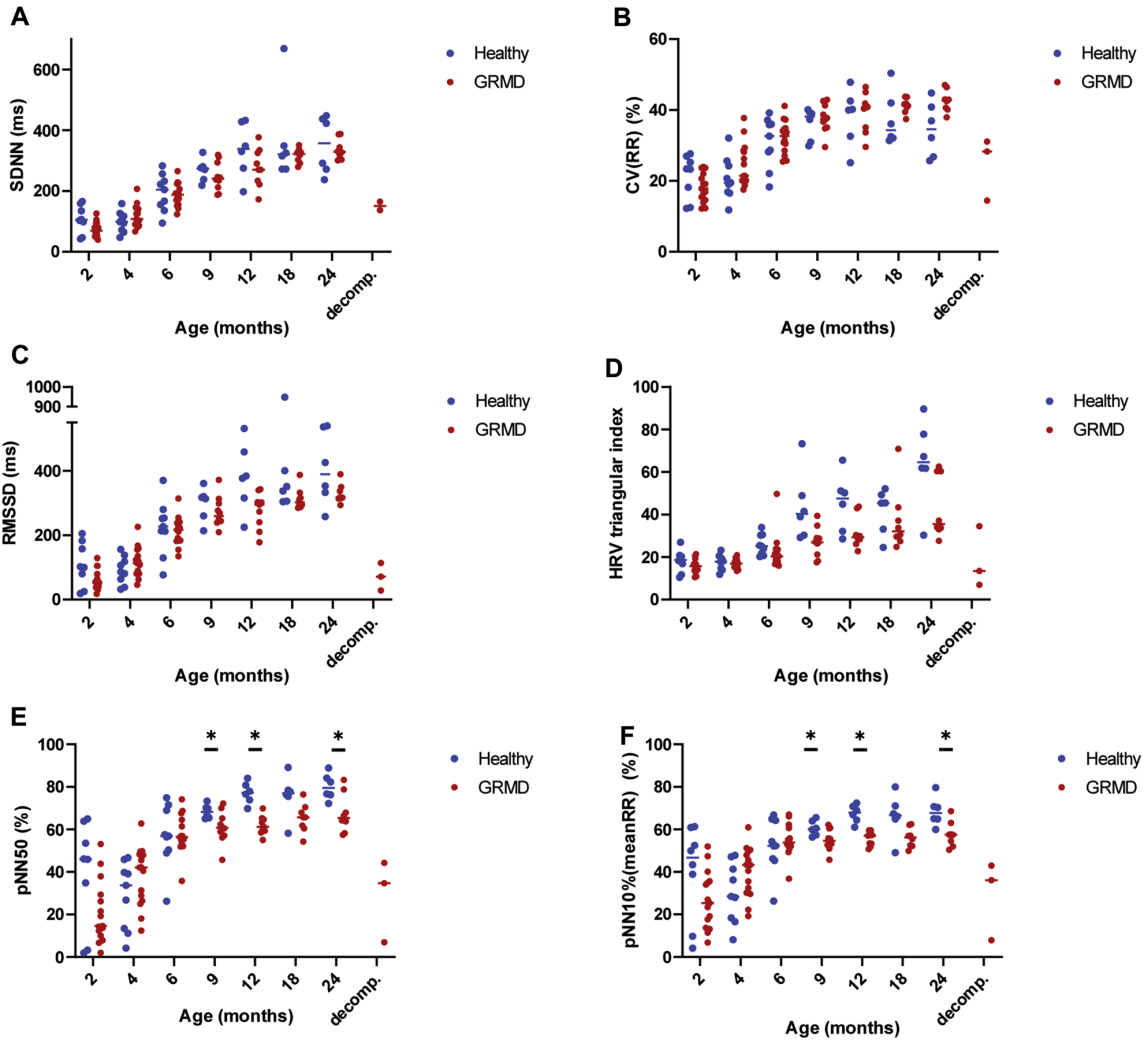
Heart rate variability—Time-domain analysis. On all the graphs, the evolution of GRMD dogs’ values are represented by red dots, and healthy dogs’ values by blue dots. A line indicates the median of each population at each timepoint. Asterisks symbolize significant differences between GRMD and healthy dogs at a given timepoint (post-hoc Fisher LSD test *p* < 0.05). (**A**) SDNN (standard deviation of the RR intervals) (**B**) CV(RR) (coefficient of variation of the RR intervals) (**C**) RMSSD (square root of the mean squared differences of successive RR intervals). (**D**) HRV triangular index. (**E**) pNN50 (percentage of interval differences of successive RR intervals of more than 50 ms duration) (**F**) pNN10%(meanRR) (percentage of interval differences of successive RR intervals of more than 10% of the mean RR duration).

**Time-domain analysis** parameters all significantly increased with age in both groups, signing an increase in HRV with growing in dogs (Fig. 3). No difference was observed between groups for SDNN (SDNN: Standard Deviation of the time between two consecutive R-waves) and CV(RR) (Coefficient of variation of RR interval, i.e. the time between two consecutive R-waves). A significant difference in RMSSD (square root of the mean squared differences of successive RR intervals), HRV triangular index, pNN50 (percentage of interval differences of successive RR intervals of more than 50 ms), and pNN10%(mean RR) (percentage of interval differences of successive RR intervals of more than 10% of the mean RR) was found between GRMD and healthy dogs’ groups over the 2–24 months period. Post-hoc tests revealed significant decrease in pNN50 and pNN10%(meanRR) at 9, 12 and 24 months of age, and the decrease was nearly significant at 18 months of age (*p* = 0.055), as well as at several timepoints from the age of 9 months for the HRV triangular index, signing decreased heart rate variability. For all the time domain analysis parameters, a drop accompanied the decompensated heart failure.

**Figure 4:**
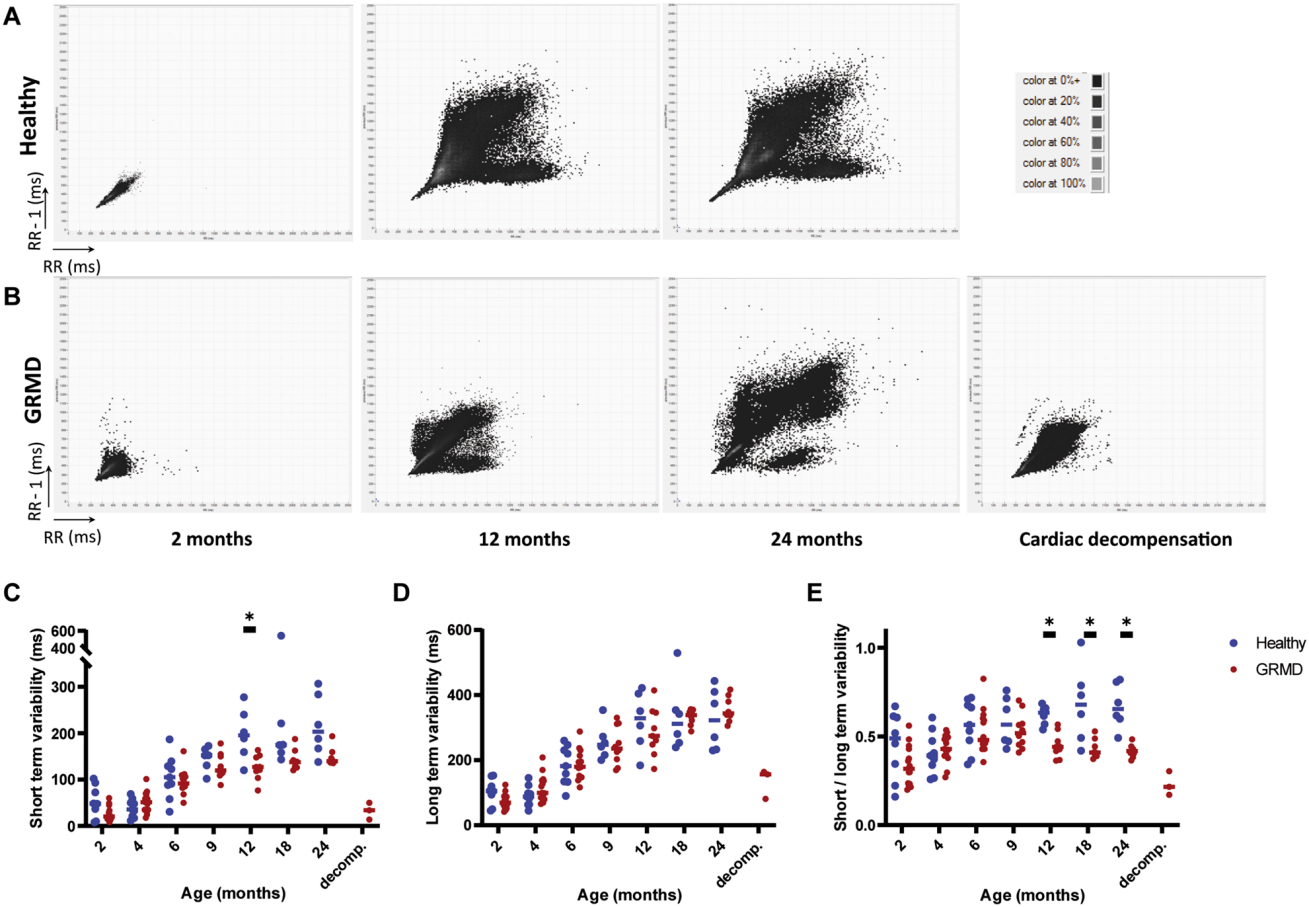
Heart rate variability—Poincaré plot analysis. (**A**) and (**B**) show typical evolution of Poincaré plots with age in a healthy and a GRMD dog, respectively. Poincaré plots were constructed by plotting each RR interval to the previous one. The scale was set at 2500 ms × 2500 ms for each graph. Examples at 2, 12, 24 months of age, and at cardiac decompensation for the GRMD dog. The dispersion of the dots on the plot increased with age, and the typical Poincaré plot aspect described in dogs was found at adulthood. The high-density zone was more condensed in GRMD dogs than in healthy dogs and the arms of the Y seemed less dense. At time of decompensated heart failure in GRMD dogs, this typical Y aspect was completely lost. (**C**) Short-term variability (STV). (**D**) Long-term variability (LTV) (**E**) STV/LTV ratio.

**Poincaré plot analysis** The visual assessment of the plots evidenced the typical branched dispersion already described in adult dogs^32,33^ (Fig. 4). The longitudinal follow-up performed in this study evidenced that this branched dispersion of the Poincaré plot was not found in puppies and installed with age. In GRMD dogs, the high-density zone near to the line of identity seemed less extended as well as the overall dispersion of the points. Decompensated heart failure in GRMD dogs was associated with a striking modification of the Poincaré plot with a remarkably condensed cloud.

Quantitative analysis was performed by calculating short-term and long-term variabilities (STV and LTV), as well as their ratio. Both STV and LTV significantly increased with age in both groups, and both STV and the STV/LTV ratio were significantly decreased in GRMD dogs compared to healthy dogs over the 2–24 months period. The STV/LTV ratio was found significantly decreased in GRMD dogs from the age of 12 months. Decompensated heart failure was associated with STV, LTV and STV/LTV decrease.

**Figure 5:**
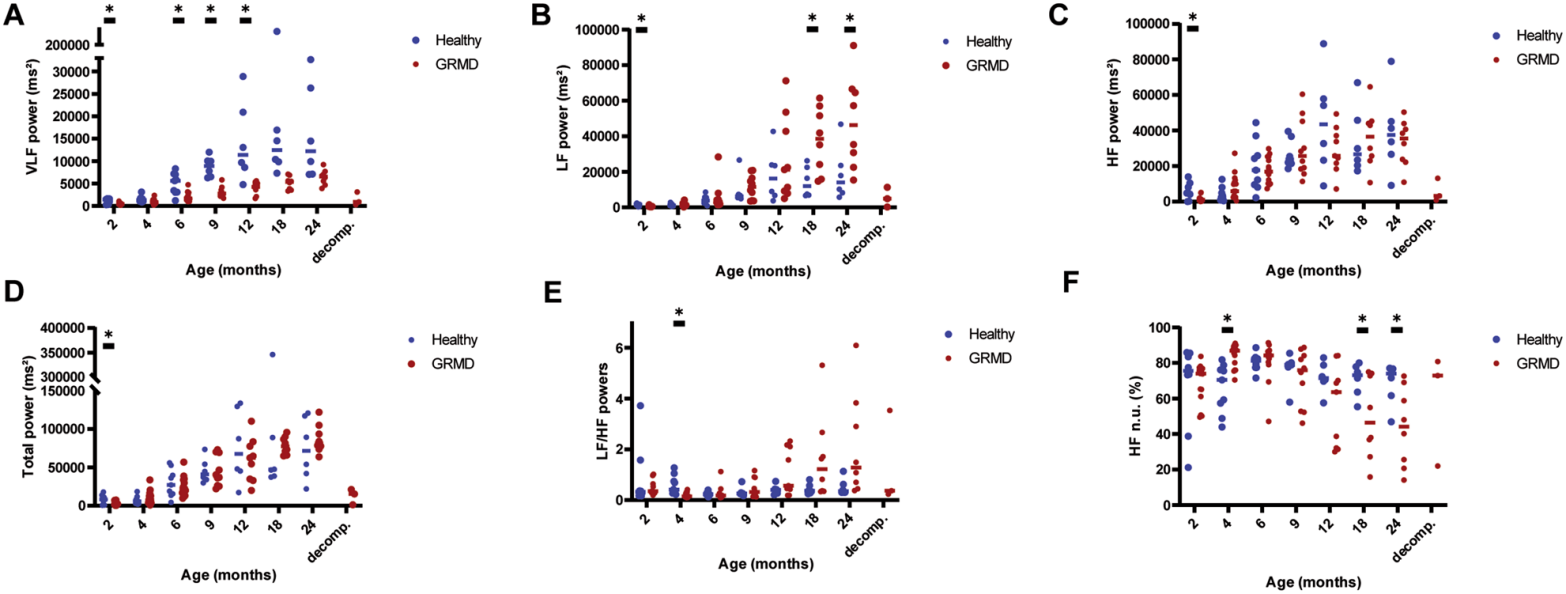
Heart rate variability—Time–frequency analysis. On all the graphs, the evolution of GRMD dogs’ values are represented by red dots, and healthy dogs’ values by blue dots. A line indicates the median of each population at each timepoint. Asterisks symbolize a significant difference between GRMD and healthy dogs at a given timepoint (post-hoc Fisher LSD test *p* < 0.05). (**A**) Very low frequency (VLF) power. (**B**) Low frequency (LF) power. (**C**) High frequency (HF) power. (**D**) Total power. (**E**) Low Frequency/High Frequency (LF/HF) ratio (**F** High frequency power, expressed in normalized units (HF n.u. = HF/(LF + HF)).

**Frequency-domain analysis** Very low frequency (VLF), low frequency (LF) and high frequency (HF) power components, as well as the total power, increased with age in both groups, and a significant difference between GRMD and healthy dogs was evidenced for VLF and LF over the 2–24 months period (Fig. 5). Post-hoc analyses showed that at the age of 2 months, the total power was significantly decreased, as well as all three components. The total power was not significantly different between groups thereafter. The LF power was only significantly higher in GRMD dogs from the age of 18 months. The HF power component was not different between groups at any age (except the 2 month-timepoint). Conversely, the VLF power component was lower in GRMD dogs than in healthy dogs from the age of 2 months and at most of the timepoints thereafter. The VLF power decreased in dogs with decompensated heart failure. It was also the case for the two other power components and the total power. The LF/HF ratio, which can be used to evaluate the sympatho-vagal balance, significantly increased with age in GRMD dogs (*p* < 0.001), but not in healthy dogs. At the age of 4 months, GRMD dogs exhibited significantly lower LF/HF values relative to healthy dogs, but this tendency rapidly inverted with some GRMD dogs exhibiting high LF/HF values. From the age of 12 months, this difference was nearly significant (*p* < 0.10). Consistently, HF expressed in normalized units (HF n.u.) was higher than in healthy dogs at 4 months of age (*p* = 0.014), and then decreased to become significantly lower from the age of 18 months.

### Serum biomarkers

Some usual cardiac serum biomarkers were measured in the dogs of this study (Fig. S5). The cardiac troponin I (cTnI) values were significantly increased in GRMD dogs at 4 months of age and tended to be higher than in healthy dogs thereafter, though not reaching statistical significance, except at the age of 24 months. At the individual level, this biomarker appeared to follow a fluctuating evolution with transient peaks and return to lower values. The NT-proBNP values, after a slight increase in some GRMD dogs at 2 months, were within those of healthy dogs until 12 months of age, when some GRMD dogs began to exhibit increased values. The difference relative to healthy dogs became significant at 12 months, and very high values of NT-proBNP were reached in ageing GRMD dogs^28^.

### Sequence of ECG changes relative to serum biomarkers and available echocardiography data

Taking together the results of this study and of a previous one focusing on echocardiography on the same animals as those used in the present study^25^, the sequential evolution of the onset of abnormalities in the occult phase of the dystrophin-deficient cardiomyopathy may thus be drawn. Changes in early echocardiographic indices such as the endo-epicardial gradient of velocity^25^, together with the VLF power component on the ECG spectral analysis and increased HR are the first hallmarks of dystrophin-deficient cardiomyopathy. Then, in the following months, but still preceding the drop of LVFS^25^, QTcV and cTnI increase, HRV decreases, sympatho-vagal balance modifies, NT-proBNP increases, and arrhythmic events aggravate. Finally, shortened PR interval and decreased LVFS^25^ occur at 24 months of age, indicating the development of dilated cardiomyopathy in young adult dogs.

### Relevance of the ECG indices to LV dysfunction

**Figure 6:**
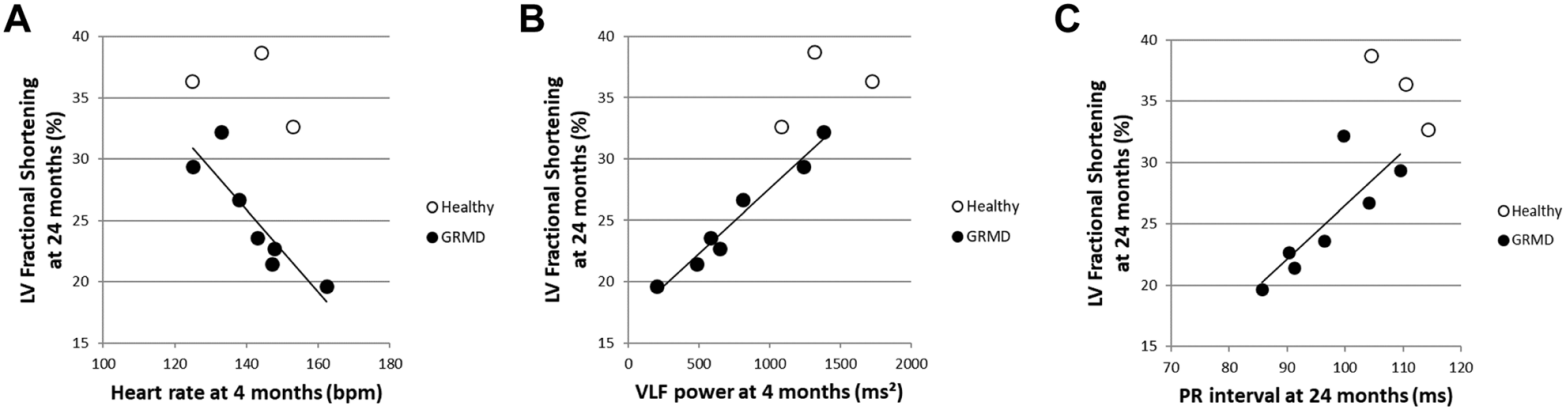
Correlations between ECG indices and fractional shortening at the age of 24 months. Some of the most significant correlations found between ECG indices at any age and the LVFS at 24 months of age in GRMD dogs (black dots) are represented on these graphs; healthy dogs’values are represented by empty dots but were not included in the correlation analysis. (**A**) Significant negative correlation between the heart rate at 4 months of age and LVFS at 24 months of age (R = − 0.786) (**B**) Significant positive correlation between the VLF power at 4 months and LVFS at 24 months of age (R = − 0.929) (**C**) Significant positive correlation between the PR interval and LVFS at 24 months of age (R = 0.738).

To understand which parameters could be related to the onset and severity of dilated cardiomyopathy, the correlation between the values obtained for the different ECG and HRV indices proven relevant in GRMD dogs at different ages, and their LVFS at the age of 24 months, previously published^25^, was studied using a Spearman rank test (n = 8 dogs). The age of 24 months was targeted for LVFS because it was previously shown to start being significantly decreased at this timepoint, with values under the lower limit of the reference interval^25^. The endo-epicardial gradient of velocity (also measured in the context of a previously published study^25^), as well as the cTnI and the NT-proBNP values were also included in the analysis. Several significant correlations were found between LVFS at 24 months and some ECG parameters measured at the age of 4 months. Notably, a negative correlation between HR at 4 months and LVFS at 24 months was found (R = − 0.786). A positive correlation between the VLF power at the age of 4 months and the LVFS at 24 months was also found (R = 0.929) (Fig. 6). Both HR and VLF were also correlated with the 24 months LVFS at the age of 12 months (R = − 0.905 and R = 0.762, respectively). Probably partly linked to the correlation with HR, pNN10%(meanRR) and pNN50 at 4 months also significantly correlated with LVFS at 24 months (R = 0.762, R = 0.857, respectively). This should however be tempered by the fact that at this timepoint these latter indices were not significantly different between GRMD and healthy dogs. In the same way, a correlation was found between LVFS at 24 months of age and the STV/LTV ratio at 6 months of age (R = − 0.738), at a stage when this ratio did not differ between GRMD and healthy dogs. A positive correlation was found between cTnI values measured at 6 and 18 months of age and the LVFS value at 24 months of age (R = 0.786 and R = 0.714, respectively). The frequency of PVBs at 24 months was negatively correlated with the 24 months LVFS (R = − 0.718). At last, the PR interval, significantly shorter relative to healthy dogs at the age of 24 months, was significantly correlated with LVFS at this timepoint (R = 0.738). This correlation was also found at 4 and 12 months of age (R = 0.929 and R = 0.762, respectively), however the values were not different from the values of healthy dogs at these earlier timepoints.

## Discussion

This study is the first one to report on longitudinal Holter-ECG findings and to investigate HRV in GRMD dogs. It reveals several similarities with ECG abnormalities found in DMD patients, and reinforces the interest of the GRMD dog as a model for dystrophin-deficient cardiomyopathy. Like in DMD patients, HR was increased, PR interval shortened, QTc prolonged, and severe ventricular arrhythmias that could cause sudden death were evidenced^9–11^. Also as described in DMD patients, most of these abnormalities were detected months before the decrease of LVFS. These early hallmarks of the dystrophin-deficient cardiomyopathy can be helpful in evaluating and translating therapeutic effects of preclinical trials performed during the occult phase of the cardiomyopathy.

The origin of the changes observed in wave intervals in DMD patients and confirmed in GRMD dogs are not fully known. However, the elongated QT, known to be a pro-arrhythmogenic ECG abnormality, was found in the dog model at a relatively early stage preceding myocardial fibrosis, and could be attributed to repolarization lengthening. It has been shown that diastolic function is early impaired both in DMD patients and in GRMD dogs, and that leakage of calcium from RyR2 channels during diastole occurs in young GRMD dogs^25,34^. Moreoever, pharmacologically stabilizing this receptor in *mdx* mice led to a normalization of the QT interval^35^, strongly suggesting that elongated QT is linked to impaired calcium handling occurring in the dystrophin-deficient myocardium. Contrarily to the QTc, the PR interval was shortened and this was a later event. The shortening of this interval in DMD has been attributed to accelerated conduction through the atrio-ventricular node, related to tissue remodeling^36^. The timeline described in our study, showing late onset of this abnormality in GRMD dogs is consistent with this hypothesis. Sympathetic activity increase in GRMD dogs could also partly account for these wave intervals abnormalities: it has been shown both to exacerbate calcium leakage from RyR2 and to facilitate atrio-ventricular node conduction.

In DMD patients, Holter-ECG is recommended in late non-ambulatory patients to detect severe ventricular arrhythmias^3^. Our study shows that ventricular arrhythmias are also commonly found in GRMD dogs, increase with age in terms of severity and correlate with cardiac dysfunction. However, importantly, severe ventricular arrhythmias could be sporadically found in young animals as well. This emphasizes the possible interest to anticipate Holter monitoring in DMD patients.

HRV was found decreased in GRMD dogs. However, the observed modifications were less numerous and striking than reported in DMD patients, using time-domain analysis^15–17,19,20^. For example, SDNN and RMSSD are decreased in DMD patients, and we did not find them modified in GRMD dogs; pNN50, HRV triangular index and STV/LTV were only modified at a few timepoints. Decompensated heart failure was however associated with striking decrease of all these indices. These few significant modifications before decompensated heart failure could be partly explained by a possible lack of sensitivity of the time-domain indices that were used to assess HRV in dogs. A recent study focused on the differences observed between dogs and humans regarding HRV. This study emphasized on the fact that pronounced respiratory sinus arrhythmia observed in dogs leads to a non-linear and non-gaussian distribution of the RR intervals, dictated by the parasympathetic system influence on the sinus node^33^. These differences could account for a possible lack of sensitivity and relevance, in GRMD dogs, of the usual linear indices used to assess HRV. Non-linear indices that also gain interest in humans, should be investigated in future studies^33,37^.

Frequency-domain analysis of the heart rate variability is a commonly used method to investigate the sympatho-vagal balance. It provides insights into oscillations of the cardiac beat-to-beat pattern, which are regulated by the activity of the autonomic nervous system^38,39^. The parasympathetic system being more rapidly acting than the sympathetic one, it is generally considered that its activity contributes to the HF band of the power spectrum, while both sympathetic and parasympathetic system activities contribute to the LF band^38,39^. This study confirmed that autonomic dysfunction in GRMD dogs resembles the one described in DMD patients. Impaired sympatho-vagal balance was evidenced by an increased LF/HF ratio and a decreased HF n.u., both suggesting a decreased vagal tone and an increased sympathetic activity, consistent with the early increase of HR. This perturbed autonomic balance has been described in young DMD patients, in whom it progresses with age, and has been proposed as a “driving force for myocardial fibrosis” and cardiac function degradation^16,20^. Some studies showed that these perturbations vary with circadian rhythm: in DMD patients, the LF/HF ratio does not decrease at night like in normal patients^15,16^. These circadian variations have not been investigated in our study, but the fact that recordings were performed at night was probably a favorable context to detect the nocturnal LF/HF elevation, progressing with age, in GRMD dogs. With age and disease evolution, some GRMD dogs exhibited very high values of LF/HF ratio and very low values of HF n.u. However, the correlation analysis did not evidence any correlation between the LF/HF ratio or HF n.u. and systolic LV function decline.

Interestingly, another component of the power spectrum, the one measured in the very low range of frequencies (VLF), was early decreased in GRMD dogs, and like HR, correlated months before with LVFS values at 24 months of age. This part of the power spectrum has never been deeply investigated in DMD patients to our knowledge, but was reported to be significantly decreased in one study^14^. Our study emphasizes the possible interest to study this part of the spectrum in DMD patients. The mechanisms underlying this VLF component have not been clearly understood, even if it is a major contributor to the total power^22^. It has been reported that VLF is related to various extra-cardiac causes, including thermoregulation, renin-angiotensin system, vasomotor tone, and physical activity^39,40^. On this latter point, a comparison of VLF values of GRMD dogs with a loss of ambulation (mean 2732.2 ms^2^ SD 1555.6 ms^2^) with those still ambulant at the age of 6 months (mean 1743.3 ms^2^ SD 1436.7 ms^2^) evidenced no difference between both subgroups. At the same timepoint, nocturnal activity measured concomitantly to the ECG, was not significantly different between the GRMD and the healthy groups of dogs. This suggests that decreased physical activity is not the main contributor to the decrease of VLF in GRMD dogs. In the context of a myopathy, thermoregulation could obviously be impacted. Core temperature was not measured during ECG recordings, but resting temperature is usually similar between GRMD dogs and healthy controls in our conditions of housing offering stable and comfort temperature theoretically requiring minimal thermoregulation efforts from dogs. We thus assume that thermoregulation is probably not either a major contributor to the VLF decrease. More recent studies show that VLF is generated intrinsically by the stimulation of afferent sensory neurons in the heart, that in turn activate feedback loops that modulate its frequency and amplitude, towards a decrease by decreasing the parasympathetic outflow and an increase by increasing the sympathetic outflow^22,41^. This new perspective offers VLF an important signification, as a direct reflect of the autonomic nervous system outflow on the intrinsic cardiac nervous system activity^22^. VLF was found to be decreased in diverse pathological contexts, probably because it is an integrative marker of autonomic-hormonal control, and it was found to be a powerful independent predictor of severe clinical events^42–46^. In the dog model of DMD, we found not only that VLF power was decreased, but also that this decrease was occurring as one of the earliest ECG modifications, with a correlation to the subsequent cardiac dysfunction. This suggests that autonomic dysfunction, reflected by decreased VLF (and to a lesser extent by LF/HF and HF n.u.), is an early event in the disease course, and might be a “driving force” for cardiac dysfunction. However, it cannot be excluded that the known vasomotor defect described in GRMD dogs and hypothesized in DMD patients could also account for the VLF decrease and promote cardiac dysfunction^47,48^. If this latter hypothesis would be confirmed, it might support the early use of ACE inhibitors, known to increase VLF, to delay cardiac dysfunction in DMD patients^39,41^.

Limitations of this study include the fact that the follow-up on healthy control dogs ended at 24 months for ethical reasons (rehoming of the healthy dogs), while some GRMD dogs could be followed-up over a longer period. This has precluded group comparisons at late stages of the cardiac disease. Another limitation is the fact that no evaluation of the deep Q waves could be performed. Deep Q waves have been previously shown to be a hallmark of the GRMD ECG^26,30^ and are commonly found in DMD patients as well^9,10^. Therefore, their evaluation would have been of great interest in this study. This was precluded by the ambulatory recording method on freely moving dogs, through skin electrodes which position can vary upon the position of the dogs leading to inconsistent wave amplitudes values. Such a wave ratio should probably be quantified in standardized conditions only permitted by standard ECG. The other limitations are associated with the correlation study. First, the fact that conditions of data acquisition differed between Holter-ECG (continuous acquisition overnight) and echocardiography (short acquisition by day) could be confusing in such analysis. Importantly, the probable strong interplay between ECG/HRV variables implies the risk to detect biased correlations and therefore a more robust statistical approach, based on multivariate regression, but precluded by the low number of animals available in this study, should have been preferred to investigate this question.

## Conclusion

This first longitudinal life-long Holter ECG study ever performed in GRMD dogs confirmed that GRMD dogs develop several ECG abnormalities reproducing the ones described in DMD patients, and provide better knowledge on their timeline. They include increased heart rate, prolonged QT and shortened PR intervals and ventricular arrhythmias that could be life-threatening. This study also provided the first investigation of the heart rate variability in this dog model, confirming that the same autonomic impairments as in DMD boys occur. The main finding of this HRV study was a decreased very low frequency power, which was found to occur at a young age, and to correlate with further left ventricular dysfunction. This part of the RR-power spectrum, described as a powerful predictor of poor outcome in various pathological conditions^42–46^, has never been specifically investigated in DMD patients; our study thus provides a preclinical rationale to promote clinical investigation of this promising marker that could have a prognostic value in DMD patients like in the canine model, and that could help improving the cardiac management of young DMD boys.

## Material and methods

### Animals

All procedures were performed in accordance with the EU Directive 2010/63/EU for animal experiments and ARRIVE guidelines, and were approved by the Ethical committee of EnvA, ANSES and UPEC under the approval numbers 20/12/12-18 and 2020-02-04-07 (APAFiS nbr #23763-2019112814263892 v7).

Fifteen GRMD dogs were included at the age of two months after genotyping as previously described^49^. The recruitment spanned over three years (March 2014 to March 2017), and the dogs were then followed-up their whole life long. When necessary, the GRMD dogs were treated for complications of the disease (mainly antibiotics in case of pneumonia, gastrostomy tube feeding in case of pronounced dysphagia), but no steroid nor other known interfering drug (*e.g.*, ACE inhibitors, β-blockers) were prescribed. Nine healthy littermates housed and cared in the same conditions, recruited from March 2014 to August 2020, were used as controls, and were followed-up until either 6 months of age (n = 3) or 24 months of age (n = 6), before being rehomed. All these dogs were part of a multi-parametric natural history study, including echocardiograms performed at the same timepoints and within few days from the Holter recordings. Detailed methods and results from this cardiac ultrasound study were reported in a recently published study^25^.

### Holter ECG-recordings

Ambulatory six-lead Holter ECG were recorded overnight in normal housing conditions using a telemetry device (EmkaPACK 4G, Emka technologies, Paris, France), allowing for 12 h-recordings. Electrodes were clipped on four skin adhesive patches placed at standardized positions after shaving: LA electrode was positioned on the left at the level of the apex beat, and RA symmetrically on the right side; RL and LL electrodes were respectively positioned in the alignment of RA and LA, at the level of the umbilicus. Electrodes wires were connected to an emitter, which was placed in a dedicated jacket worn by freely moving dogs. ECG was acquired at a sampling rate of 500 Hz using the software IOX (Emka technologies).

Iterative recordings were performed at the following ages: 2, 4, 6, 9, 12, 18, 24 months of age. In surviving GRMD dogs, recordings were also performed at 36, 48 and 60 months of age. A recording was also performed at time of decompensated heart failure for 3 of the GRMD dogs (respectively at 63, 65 and 78 months of age). The number of dogs recorded at each age is available in Tables 1 and S1. Figures in the main paper represent the data from the 2 to 24 months period. The values of GRMD dogs measured at later timepoints are illustrated in Fig. S3.

### ECG analysis

Quantitative ECG analysis was performed in the software ecgAUTO (Emka technologies), on the lead II. All the analyses were performed by the same operator (IB), a doctor in veterinary medicine, who received training in canine cardiology and electrocardiography within her veterinary medicine initial education. For each trace, a waveform library including normal beats and possible arrhythmic events was built manually by scanning each trace, and positioning markers for each wave, to allow reliable recognition of beats and waves by the software. Automatic library-based analysis was then run, and the following parameters were calculated and averaged over the whole trace: heart rate (HR (beats/min)), PR interval (ms), QT interval (ms). In order to compensate for HR variations among dogs and ages, the best QT correction formula to be applied to this cohort was evaluated in a preliminary study based on the data obtained in the healthy dogs, as recommended in the literature^31^. The following corrections were assessed: Bazett’s QT correction (QTcB), Friedericia’s QT correction (QTcF), Van de Water’s QT correction (QTcV), and Matsunaga’s QT correction (QTcM)^31^. The correction formula which was the best in decorrelating QT values from heart rate was kept for the subsequent analysis. The Q/R waves amplitude ratio was calculated to detect possible deep Q-waves in GRMD dogs as previously described^26,30^. However, the analysis of this ratio over the Holter ECG traces revealed inconsistencies: in a given dog trace (including healthy dogs), Q waves could be found deep at some periods and normal at some other periods suggesting possible position-related change of ECG morphology. Considering that recordings on freely-moving dogs may not be the adequate experimental setting to assess wave-size ratios, we decided not to include these data in the study.

The arrhythmic events were visually characterized, categorized and their number was quantified. Concerning premature ventricular beats (PVBs), their frequency was expressed as a number of PVBs per hour. The number of doublets, triplets or salvos was quantified, and each trace was stratified according to the Lown classification of PVBs severity (0: no PVB; 1: rare isolated PVBs < 30/hr; 2: frequent isolated PVBs > 30/hr; 3a: isolated polymorphic PVBs; 3b: bigeminated PVBs; 4a: doublets or triplets; 4b: ventricular tachycardia (> 3 consecutive PVBs); 5: R/T phenomenon)^50^.

The presence or absence of fragmented QRS was assessed visually on the traces by a single observer who was not blinded to disease status of the dogs.

### Heart rate variability (HRV) analysis

The HRV analysis was performed using the software ecgAUTO (Emka technologies) after exclusion of the RR intervals preceding and following each arrhythmic beat to keep only RR intervals between normal complexes in the analysis.

Time-domain analysis was first performed providing classical HRV indices, including SDNN (standard deviation of the RR intervals), CV(RR) (coefficient of variation of the RR intervals, i.e. the SDNN/meanRR ratio), RMSSD (square root of the mean squared differences of successive RR intervals), HRV triangular index (total number of RR divided by the number of RR on the highest bin of the RR-density histogram constructed after a 128 Hz sampling), and pNN50 (percentage of interval differences of successive RR intervals of more than 50 ms)^23^. A pNN10% (mean RR) was also calculated as the percentage of interval differences of successive RR intervals of more than 10% of the mean RR on the trace, to adapt to differences in HR between ECGs, groups and ages.

Poincaré plot analysis was performed by plotting each RR to the previous one. Qualitative visual analyses of the plots were performed, and associated with a quantitative analysis: short-term and long-term variabilities (STV and LTV, also commonly called SD1 and SD2) were calculated. LTV was the standard deviation of the points along the RR = RR-1 line (line of identity), and STV was the standard deviation of the points along a perpendicular crossing this line of identity at the mean RR point value^32^.

Frequency-domain analysis of the RR intervals was performed after 512-points Fast Fourier Transform on 15-min-long epochs and a rectangular windowing, allowing the calculation of the Very Low, Low and High Frequency powers (VLF, LF and HF) respectively corresponding to the areas under the curve of the spectrum within the following frequency bands: 0.003–0.04 Hz, 0.04–0.15 Hz and 0.15 Hz-0.4 Hz respectively. The mean LF/HF ratio across the epochs, and the mean HF n.u. (normalized units, HF n.u. = HF/(LF + HF)) were calculated and used in the analysis^23^.

### Serum biomarkers

Blood samples were collected from the dogs at the same timepoints as the ECG recordings. Troponin I (cTnI) was measured using the dedicated kit for the Immulite 2000 analyzer (reference L2KTI; Siemens Healthcare, Malvern, PA). NT-proBNP was measured according to a patented method (Cardiopet proBNP), by Idexx laboratories, France.

### Statistical analysis

The best QT correction formula was assessed by calculating a Pearson coefficient of correlation between QTc and HR on a healthy dogs’ dataset for which normality of distribution was checked using a Shapiro–Wilk test (*p* > 0.05), and by determining the slope of the regression line.

A repeated measures ANOVA was used to assess the age effect (within effect) in each group (GRMD, Healthy). The overall group (GRMD vs healthy) effect was assessed using a mixed effects model followed by a Fisher’ LSD post-hoc test to assess group effect at each time point.

Correlation between the indices demonstrated to be different in GRMD dogs relative to healthy dogs at any age, and left ventricular fractional shortening (LVFS) measured during the echocardiogram performed at the age of 24 months was assessed in the 8 surviving GRMD dogs, using a Spearman coefficient of correlation. LVFS data were collected in the context of an echocardiography study performed on the same dogs and recently published^25^. The level of statistical significance was set at *p* ≤ 0.05.

### Supplementary Information

Supplementary Information 1.

Supplementary Information 2.

Supplementary Information 3.

Supplementary Information 4.

Supplementary Information 5.

Supplementary Legends.

## Data Availability

The data that support the findings of this study are available from the corresponding author, upon reasonable request.

## Abbreviations

cTnI: Cardiac troponin I
DMD: Duchenne muscular dystrophy
ECG: Electrocardiogram
GRMD: Golden retriever muscular dystrophy
HF: High frequency
HR: Heart rate
HRV: Heart rate variability
LF: Low frequency
LGE: Late gadolinium enhancement
LTV: Long-term variability
LVEF: Left ventricular ejection fraction
NT-proBNP: N-terminal part of the pro-brain natriuretic peptide
pNN50: Percentage of interval differences of successive RR intervals of more than 50 ms
pNN10% (meanRR): Percentage of interval differences of successive RR intervals of more than 10% of the mean RR
QTc: Corrected QT interval
QTcB: Bazett’s corrected QT interval
QTcF: Friedericia’s corrected QT interval
QTcM: Matsunaga’s corrected QT interval
QTcV: Van de Water’s corrected QT interval
RMSSD: Square root of the mean squared differences of successive RR intervals
RR interval: Time between two successive R-waves
SDNN: Standard deviation of the RR intervals
LVFS: Left ventricular fractional shortening
STV: Short-term variability
VLF: Very low frequency
PVB: Premature ventricular beat
VT: Ventricular tachycardia
