# Supplementary figures and images for: Ambulatory electrocardiographic longitudinal monitoring in a canine model for Duchenne muscular dystrophy identifies decreased very low frequency power as a hallmark of impaired heart rate variability
Source: Sci Rep. 2024 Apr 18;14:8969. doi: 10.1038/s41598-024-59196-z (PMC11026469; doi:10.1038/s41598-024-59196-z)

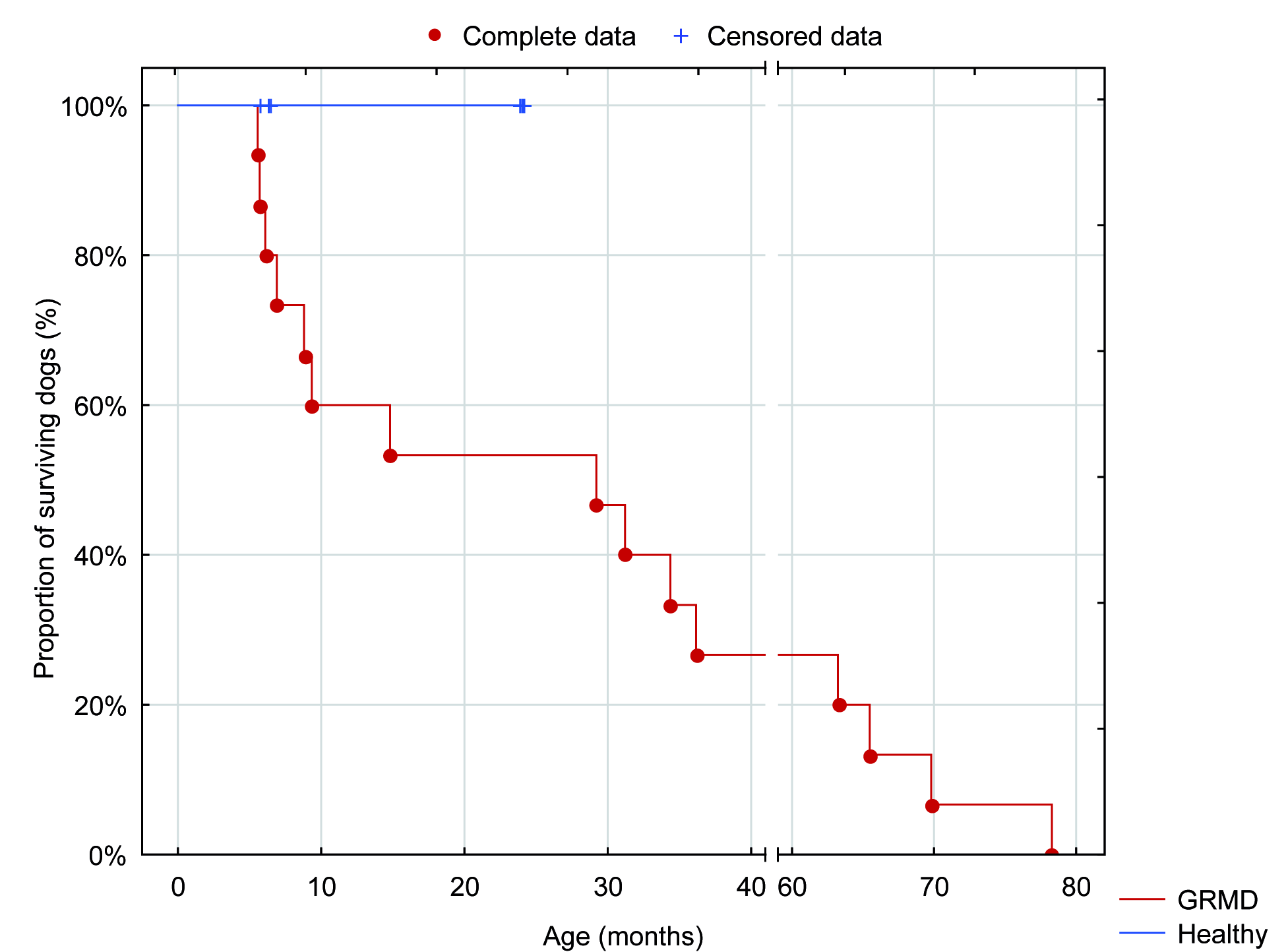

Supplement: Supplementary file 1 — Supplementary Information 1. [file 41598_2024_59196_MOESM1_ESM.tif]

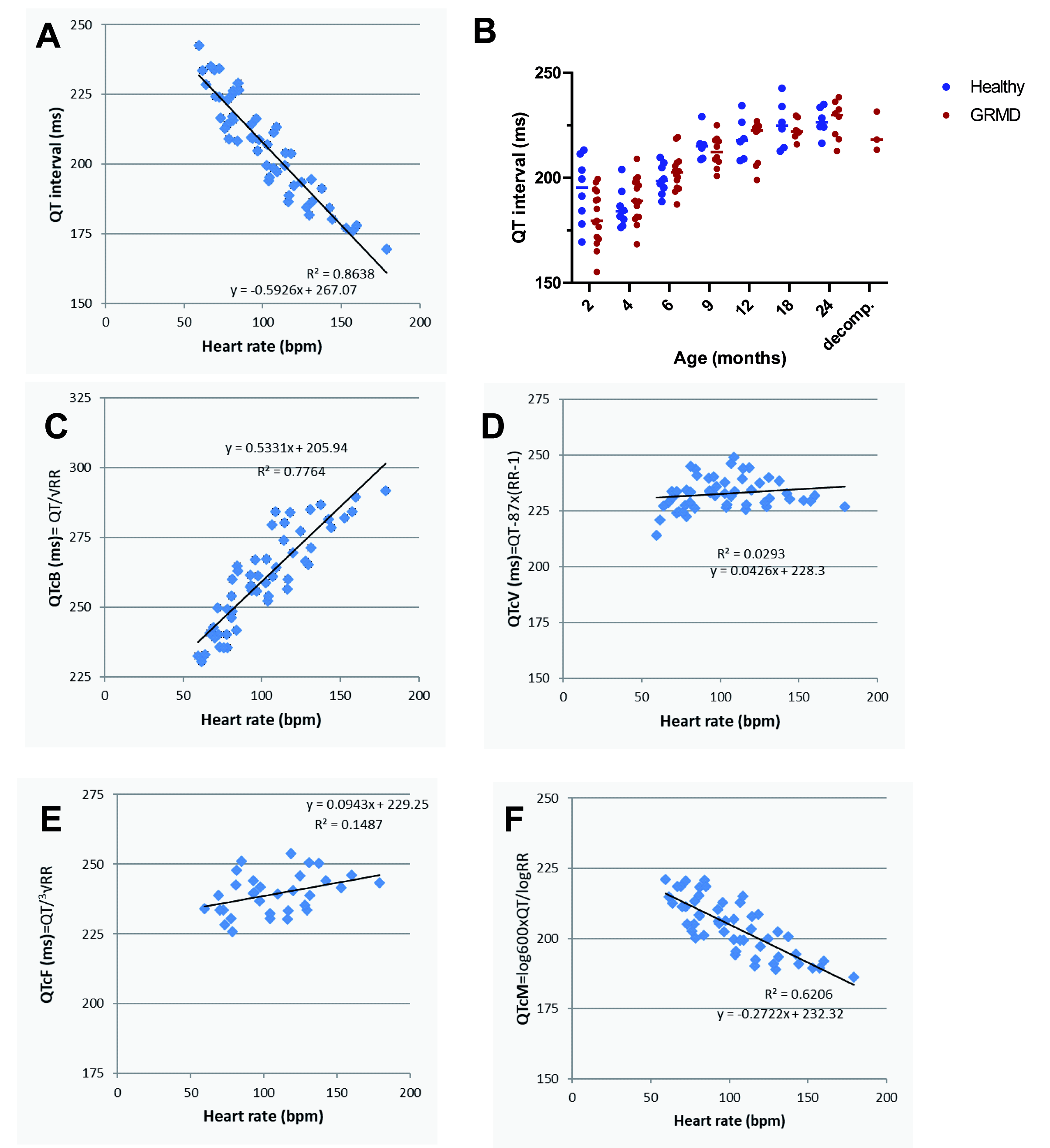

Supplement: Supplementary file 3 — Supplementary Information 3. [file 41598_2024_59196_MOESM3_ESM.tif]

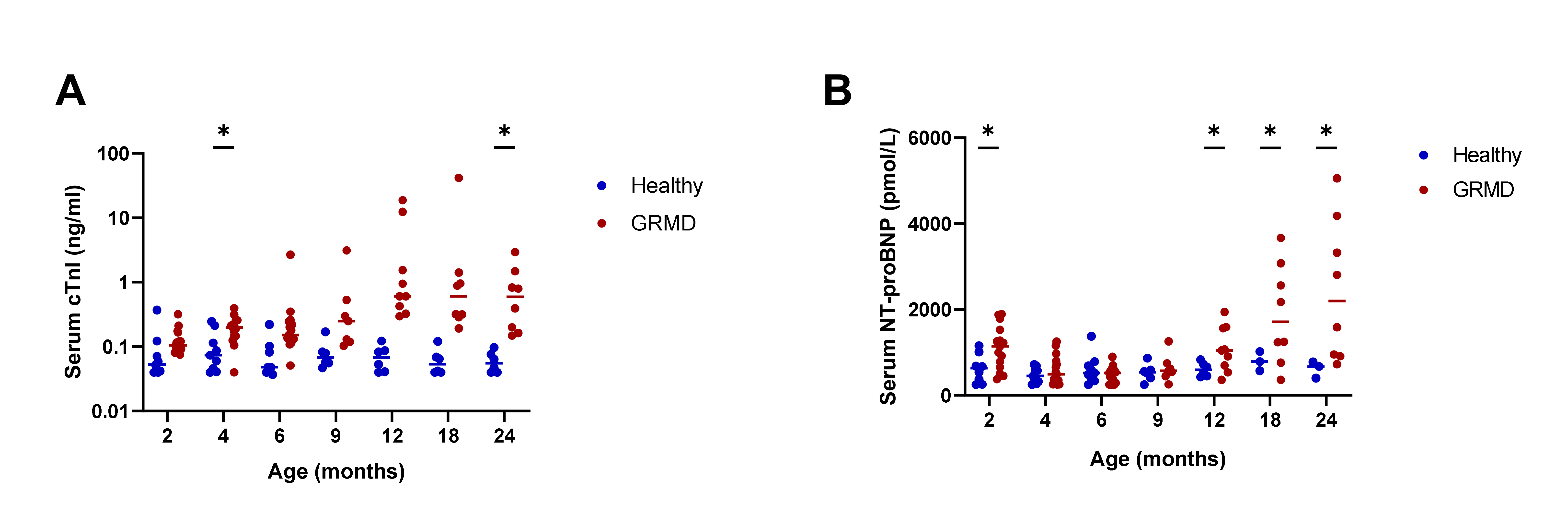

Supplement: Supplementary file 5 — Supplementary Information 5. [file 41598_2024_59196_MOESM5_ESM.tif]
