# Supplementary material for: Ambulatory electrocardiographic longitudinal monitoring in a canine model for Duchenne muscular dystrophy identifies decreased very low frequency power as a hallmark of impaired heart rate variability
Source: Sci Rep. 2024 Apr 18;14:8969. doi: 10.1038/s41598-024-59196-z (PMC11026469; doi:10.1038/s41598-024-59196-z)
Supplement: Supplementary file 2 — Supplementary Information 2. [file 41598_2024_59196_MOESM2_ESM.docx]

Table S1 Detailed results

|  | **Age (months):** | **2** | **4** | **6** | **9** | **12** | **18** | **24** | **36** | **48** | **60** | Decompensation | Age effect (2-24 months) | Group effect (2-24 months) |
| --- | --- | --- | --- | --- | --- | --- | --- | --- | --- | --- | --- | --- | --- | --- |
|  | Healthy n= | 8 | 9 | 9 | 6 | 6 | 6 | 6 | - | - | - | - |  |  |
|  | GRMD n= | 15 | 15 | 15 | 10 | 9 | 8 | 8 | 5 | 4 | 4 | 2 |  |  |
| HR (bpm) | Healthy, mean (SD) | 133.4 (26.2) | 136.4 (14.2) | 105.8 (8.6) | 90.8 (6.5) | 79.4 (4.0) | 74.2 (8.8) | 68.0 (4.6) | - | - | - | - | **<0.001** | **<0.001** |
|  | GRMD, mean (SD) | 156.6 (19.4) | 134.8 (11.4) | 114.4 (9.2) | 104.7 (10.7) | 99.5 (7.4) | 91.7 (5.7) | 90.1 (10.1) | 90.5 (15.0) | 93.3 (6.8) | 91.3 (10.7) | 128.3 (7.6) | **<0.001** |  |
|  | Fisher LSD p-value | **0.049** | 0.781 | **0.032** | **0.006** | **<0.001** | **0.003** | **<0.001** | - | - | - | - |  |  |
| QT (ms) | Healthy, mean (SD) | 193.9 (15.8) | 185.4 (8.7) | 199.2 (6.9) | 215.6 (7.4) | 219.0 (10.1) | 225.6 (11.5) | 227.0 (6.9) | - | - | - | - | **<0.001** | 0.566 |
|  | GRMD, mean (SD) | 181.1 (13.1) | 190 (10.8) | 202.6 (8.6) | 212.5 (7.5) | 217.4 (10.4) | 222.8 (4.5) | 227.3 (9.1) | 230.1 (10.2) | 228.1 (10.1) | 229.5 (9.0) | 215.8 (3.4) | **<0.001** |  |
|  | Fisher LSD p-value | 0.074 | 0.265 | 0.302 | 0.442 | 0.778 | 0.596 | 0.937 | - | - | - | - |  |  |
| QTcV (ms) | Healthy, mean (SD) | 238.6 (7.7) | 232.9 (6.0) | 232.4 (3.2) | 237.7 (4.2) | 230.1 (8.3) | 229.1 (9.3) | 226.1 (3.6) | - | - | - | - | **0.006** | **0.002** |
|  | GRMD, mean (SD) | 230.9 (12.2) | 235.9 (8.6) | 239.4 (7.2) | 242.6 (4.1) | 243.7 (8.4) | 243.2 (5.7) | 245.1 (7.9) | 247.4 (6.2) | 245.7 (8.9) | 247 (4.5) | 258.7 (0.5) | **<0.001** |  |
|  | Fisher LSD p-value | 0.076 | 0.335 | **0.004** | **0.046** | **0.010** | **0.012** | **<0.001** | - | - | - | - |  |  |
| PR (ms) | Healthy, mean (SD) | 75.4 (10.8) | 77.7 (6.4) | 87.7 (9.4) | 99.1 (11.7) | 101.5 (8.5) | 107.5 (11.5) | 110.9 (8.6) | - | - | - | - | **<0.001** | **0.035** |
|  | GRMD, mean (SD) | 71.5 (8.4) | 77.4 (8.3) | 82.3 (8.2) | 92.2 (9.3) | 95.9 (8.0) | 98.4 (8.2) | 98.0 (8.6) | 104.9 (10.3) | 95.3 (12.1) | 99.1 (11.5) | 94.4 (9.2) | **<0.001** |  |
|  | Fisher LSD p-value | 0.396 | 0.909 | 0.175 | 0.252 | 0.231 | 0.134 | **0.018** | - | - | - | - |  |  |
| PVBs Frequency (PVBs/hour) | Healthy, mean (SD) | 0.2 (0.7) | 3.0 (8.9) | 12.6 (36.4) | 8.5 (17.6) | 42.7 (65.4) | 33.8 (56.1) | 36.5 (72.4) |  |  |  |  | 0.280 | 0.081 |
|  | GRMD, mean (SD) | 0.00 (0.00) | 3.1 (12.0) | 6.6 (22.3) | 5.0 (7.5) | 12.6 (32.0) | 196.9 (285.4) | 270.4 (380.2) | 156.7 (221.7) | 183.6 (213.0) | 129.6 (144.5) | 71.5 (101.4) | **0.006** |  |
|  | Fisher LSD p-value | 0.351 | 0.975 | 0.659 | 0.652 | 0.331 | 0.155 | 0.129 |  |  |  |  |  |  |
| SDNN (ms) | Healthy, mean (SD) | 106.9 (45.9) | 98.0 (34.1) | 194.5 (61.0) | 268.4 (37.6) | 335.0 (90.3) | 367.2 (150.9) | 351.3 (94.7) | - | - | - | - | **<0.001** | 0.098 |
|  | GRMD, mean (SD) | 73.7 (23.6) | 117.1 (37.3) | 187.9 (37.7) | 247.1 (47.0) | 277.1 (63.0) | 316.3 (24.0) | 337.3 (33.9) | 331.2 (72.6) | 380.9 (40.8) | 356.2 (28.6) | 150.6 (19.8) | **<0.001** |  |
|  | Fisher LSD p-value | 0.088 | 0.216 | 0.778 | 0.348 | 0.208 | 0.449 | 0.741 | - | - | - | - |  |  |
| CV(RR) (ms) | Healthy, mean (SD) | 21.2 (6.2) | 20.7 (5.9) | 30.9 (7.2) | 36.1 (4.5) | 38.0 (8.0) | 37.4 (7.5) | 34.5 (7.7) | - | - | - | - | **<0.001** | 0.197 |
|  | GRMD, mean (SD) | 18.1 (4.1) | 24.5 (6.00) | 32.3 (4.51) | 37.6 (4.04) | 39.3 (5.5) | 41.2 (2.1) | 42.6 (3.1) | 41.4 (5.1) | 47.7 (4.5) | 44.8 (2.2) | 29.7 (2.0) | **<0.001** |  |
|  | Fisher LSD p-value | 0.234 | 0.148 | 0.591 | 0.501 | 0.745 | 0.274 | 0.050 | - | - | - | - |  |  |
| RMSSD (ms) | Healthy, mean (SD) | 109.0 (68.9) | 91.0 (42.7) | 223.9 (84.4) | 297.7 (52.2) | 382.1 (107.1) | 441.3 (250.8) | 407.7 (114.3) | - | - | - | - | **<0.001** | **0.009** |
|  | GRMD, mean (SD) | 59.9 (31.3) | 116.7 (46.9) | 212.9 (43.9) | 272 (46.2) | 276 (56.3) | 313.5 (33.9) | 329.4 (29.4) | 319.6 (73.7) | 328.8 (41.1) | 337.5 (53.7) | 92.4 (30.0) | **<0.001** |  |
|  | Fisher LSD p-value | 0.090 | 0.186 | 0.724 | 0.345 | 0.062 | 0.269 | 0.157 | - | - | - | - |  |  |
| pNN50 (%) | Healthy, mean (SD) | 39.3 (24.7) | 29.0 (15.9) | 56.9 (14.7) | 68.4 (3.2) | 77.1 (5.0) | 75.9 (10.0) | 80.1 (6.1) | - | - | - | - | **<0.001** | **0.008** |
|  | GRMD, mean (SD) | 20.9 (14.7) | 38.1 (13.8) | 57.7 (9.0) | 60.9 (7.5) | 62 (4.3) | 65.3 (6.7) | 67.5 (9.2) | 69.7 (8.4) | 65.9 (4.4) | 69.3 (8.6) | 39.5 (6.8) | **<0.001** |  |
|  | Fisher LSD p-value | 0.082 | 0.175 | 0.876 | **0.016** | **<0.001** | 0.055 | **0.010** | - | - | - | - |  |  |
| pNN10%(mean RR) (%) | Healthy, mean (SD) | 40.1 (21.9) | 30.2 (14.0) | 52.2 (12.6) | 60.6 (3.6) | 67.4 (4.2) | 66.4 (10.1) | 68.4 (6.8) | - | - | - | - | **<0.001** | **0.020** |
|  | GRMD, mean (SD) | 26.8 (13.6) | 40.1 (11.7) | 54.9 (7.3) | 55.1 (5.0) | 55.8 (3.5) | 56.1 (4.6) | 57.6 (5.9) | 58.1 (5.2) | 55.8 (1.8) | 58.7 (4.5) | 39.6 (4.8) | **<0.001** |  |
|  | Fisher LSD p-value | 0.148 | 0.095 | 0.617 | **0.024** | **<0.001** | 0.055 | **0.011** | - | - | - | - |  |  |
| HRV triangular index | Healthy, mean (SD) | 18.0 (5.2) | 7.4 (3.5) | 25.8 (4.8) | 43.7 (16.3) | 45.4 (13.6) | 41.6 (10.6) | 64.8 (20.0) | - | - | - | - | **<0.001** | **0.003** |
|  | GRMD, mean (SD) | 15.7 (3.1) | 17.2 (2.2) | 22.3 (8.1) | 26.9 (6.9) | 31.4 (7.2) | 37.2 (14.8) | 43.6 (14.7) | 39.5 (17.0) | 36.2 (10.9) | 37.7 (17.4) | 24.0 (14.9) | **<0.001** |  |
|  | Fisher LSD p-value | 0.280 | 0.840 | 0.206 | 0.052 | 0.053 | 0.522 | 0.057 | - | - | - | - |  |  |
| STV (ms) | Healthy, mean (SD) | 52.3 (35.0) | 38.1 (19.7) | 105.6 (46.0) | 145.7 (25.8) | 197.8 (56.1) | 235.9 (153.3) | 216.7 (66.3) | - | - | - | - | **<0.001** | **0.001** |
|  | GRMD, mean (SD) | 25.7 (14.8) | 50.5 (21.9) | 96.0 (24.6) | 127.4 (26.2) | 126.3 (26.4) | 142.6 (22.2) | 148.4 (19.7) | 153.2 (41.4) | 155.0 (19.9) | 162.8 (34.3) | 41.7 (11.3) | **<0.001** |  |
|  | Fisher LSD p-value | 0.073 | 0.171 | 0.575 | 0.198 | **0.024** | 0.197 | 0.053 | - | - | - | - |  |  |
| LTV (ms) | Healthy, mean (SD) | 102.6 (40.5) | 89.2 (31.2) | 183.7 (58.8) | 256.1 (54.3) | 321.0 (90.5) | 332.9 (106.6) | 326.7 (93.9) | - | - | - | - | **<0.001** | **0.839** |
|  | GRMD, mean (SD) | 73.5 (22.9) | 114 (39.9) | 185.3 (44.3) | 245.1 (57.3) | 286.8 (74.1) | 331.5 (24.1) | 335.7 (39.2) | 347.3 (81.7) | 417.9 (61.1) | 372.7 (36.1) | 160.0 (5.1) | **<0.001** |  |
|  | Fisher LSD p-value | 0.092 | 0.106 | 0.946 | 0.706 | 0.461 | 0.976 | 0.501 | - | - | - | - |  |  |
| STV/LTV | Healthy, mean (SD) | 0.45 (0.19) | 0.41 (0.12) | 0.56 (0.14) | 0.59 (0.14) | 0.62 (0.06) | 0.68 (0.22) | 0.67 (0.13) | - | - | - | - | **0.001** | **<0.001** |
|  | GRMD, mean (SD) | 0.33 (0.11) | 0.43 (0.08) | 0.53 (0.12) | 0.53 (0.10) | 0.45 (0.07) | 0.43 (0.05) | 0.42 (0.04) | 0.44 (0.07) | 0.38 (0.09) | 0.44 (0.09) | 0.26 (0.06) | **<0.001** |  |
|  | Fisher LSD p-value | 0.144 | 0.556 | 0.554 | 0.437 | **<0.001** | **0.038** | **0.004** | - | - | - | - |  |  |
| VLF (ms²) | Healthy, mean (SD) | 1156.6 (565.8) | 1497.3 (693.3) | 4973.7 (2369.6) | 8782.6 (2265.4) | 14323.7 (8969.4) | 52578.2 (99921.0) | 16258.1 (10777.8) | - | - | - | - | 0.271 | **0.010** |
|  | GRMD, mean (SD) | 455.3 (246.8) | 962.8 (534.5) | 2007.1 (1013.8) | 3077.7 (1202.1) | 4116.9 (1402.8) | 5135.5 (1449.1) | 6411.2 (1699.3) | 5253.3 (2079.9) | 6253.6 (1772.4) | 6641.2 (2755.7) | 2005.4 (1558.3) | **<0.001** |  |
|  | Fisher LSD p-value | **0.009** | 0.067 | **0.005** | **<0.001** | **0.038** | 0.297 | 0.075 | - | - | - | - |  |  |
| LF (ms²) | Healthy, mean (SD) | 1378.5 (733.2) | 1380.0 (740.2) | 4201.8 (2281.2) | 9471.2 (8459.4) | 18229.4 (14751.0) | 15468.8 (7600.7) | 18720.7 (15239.6) | - | - | - | - | **0.001** | **0.003** |
|  | GRMD, mean (SD) | 539.3 (354.4) | 1309.7 (945.1) | 5028.4 (6795.7) | 11476.7 (6726.8) | 27170.5 (23400.2) | 37732.4 (18307.1) | 47926.1 (25972.1) | 49968.6 (39039.5) | 75371.1 (40038.4) | 52881.7 (18309.8) | 8102.1 (4563.2) | **<0.001** |  |
|  | Fisher LSD p-value | **0.014** | 0.841 | 0.722 | 0.633 | 0.381 | **0.009** | **0.022** | - | - | - | - |  |  |
| HF (ms²) | Healthy, mean (SD) | 5836.2 (4811.8) | 3956.9 (4003.7) | 20172.9 (14020.1) | 27299.2 (8724.2) | 44279.7 (28620.1) | 33952.1 (19088.1) | 39144.0 (23290.1) | - | - | - | - | **<0.001** | 0.457 |
|  | GRMD, mean (SD) | 1561.7 (1530.0) | 8574 (6981.8) | 17660.2 (6967.4) | 30126.9 (16350.8) | 28834.3 (12939.3) | 35811.8 (16765.5) | 32883.1 (13091.0) | 31840.6 (12619.1) | 30253.6 (14889.6) | 38446.8 (18156.4) | 8150.0 (6993.0) | **<0.001** |  |
|  | Fisher LSD p-value | **0.041** | 0.052 | 0.649 | 0.659 | 0.259 | 0.853 | 0.572 | - | - | - | - |  |  |
| HF n.u. (%) | Healthy, mean (SD) | 67.4 (24.1) | 65.8 (13.8) | 80.4 (4.8) | 76.5 (9.5) | 72.0 (8.7) | 70.5 (9.4) | 68.4 (12.0) | - | - | - | - | 0.212 | 0.068 |
|  | GRMD, mean (SD) | 68.9 (11.3) | 83.9 (6.6) | 81.4 (10.8) | 71.4 (15.8) | 55.9 (22.7) | 49.3 (23.1) | 43.7 (22.3) | 48.3 (32.1) | 32.2 (21.1) | 42.1 (19.3) | 47.5 (35.9) | **<0.001** |  |
|  | Fisher LSD p-value | 0.868 | **0.004** | 0.762 | 0.435 | 0.080 | **0.042** | **0.022** |  |  |  |  |  |  |
| LF/HF | Healthy, mean (SD) | 0.86 (1.25) | 0.59 (0.38) | 0.25 (0.08) | 0.33 (0.20) | 0.41 (0.19) | 0.44 (0.21) | 0.51 (0.33) |  |  |  |  | 0.310 | **0.033** |
|  | GRMD, mean (SD) | 0.49 (0.28) | 0.20 (0.10) | 0.26 (0.25) | 0.47 (0.38) | 1.11 (0.92) | 1.65 (1.70) | 2.11 (2.02) | 2.07 (2.10) | 3.53 (2.93) | 2.26 (2.62) | 1.95 (2.24) | **<0.001** |  |
|  | Fisher LSD p-value | 0.444 | **0.014** | 0.869 | 0.327 | 0.051 | 0.085 | 0.061 |  |  |  |  |  |  |
| Total power | Healthy, mean (SD) | 8371.3 (5952.5) | 6834.2 (5340.9) | 29348.4 (18010.3) | 45553.0 (16135.2) | 76832.8 (47887.6) | 100889.9 (121555.2) | 74122.8 (41005.4) |  |  |  |  | **0.011** | 0.525 |
|  | GRMD, mean (SD) | 2556.3 (2081.7) | 10846.5 (8391.8) | 24745.6 (12481.1) | 44681.3 (19545.9) | 60121.7 (28256.8) | 78679.6 (11537.3) | 87220.4 (18761.7) | 2.07 (2.10) | 3.53 (2.93) | 2.26 (2.62) | 1.95 (2.24) | **<0.001** |  |
|  | Fisher LSD p-value | **0.028** | 0.167 | 0.511 | 0.925 | 0.465 | 0.674 | 0.492 |  |  |  |  |  |  |
