# Supplementary material for: Ambulatory electrocardiographic longitudinal monitoring in a canine model for Duchenne muscular dystrophy identifies decreased very low frequency power as a hallmark of impaired heart rate variability
Source: Sci Rep. 2024 Apr 18;14:8969. doi: 10.1038/s41598-024-59196-z (PMC11026469; doi:10.1038/s41598-024-59196-z)
Supplement: Supplementary file 6 — Supplementary Legends. [file 41598_2024_59196_MOESM6_ESM.docx]

Figure S1 : Survival curve of the dogs included in the study

Nine healthy dogs (blue line) were included and rehomed at 6 (n=3) and 24 months of age (n=6). Fifteen GRMD dogs (black line) were included. Four of them were euthanized around six months of age due to a loss of ambulation and marked dyspnea for one of them. All of them could undergo the 6 months ECG recording. Two dogs were euthanized around 9 months of age, the first one was due to a pulmonary lobar torsion, and the second one in whom the 9 months ECG recording was completed was due to poor mobility. Nine dogs were still alive at 12 months of age. A seventh dog was euthanized at the age of 15 months due to poor mobility associated with marked dyspnea. Eight GRMD dogs were still alive at the 18 and 24 months timepoints. In their third year, two dogs underwent laparotomia intending to reverse an ileus, due in both cases to entangled pylorico-duodenal junction in the hiatal hernia. The proximal part of the descendant duodenum was in both cases necrotic, and the dogs were thus euthanized during surgery. A third GRMD dog died within his third year after a failed resuscitation attempt, following a cardiorespiratory arrest at induction of an anesthesia. Five dogs were still alive at the 36 months timepoint. One of them suddenly died at the age of 36 months around one hour after uncomplicated recovery from general anesthesia during which a short syncopal episode occurred, and the resuscitation attempt failed. The four last GRMD dogs all survived until the 60 months timepoint. Three of them had a decompensated heart failure, manifesting ascites, pleural effusion, and pulmonary edema in one dog, at respectively 63, 65 and 78 months of age and justifying euthanasia. The fourth one, which was in an overall very good shape with a very mild locomotor form of the disease, had to be euthanized at 70 months of age, due to an idiopathic pericardial effusion.

Figure S2 : QT correction study

This study was based on the data obtained on healthy dogs overtime. QTc values according to different formulae (Y axis) were plotted versus HR values (X axis). A: As expected, QT was negatively correlated with HR in the healthy dogs’ dataset B: As a consequence, QT increased with age and HR decrease in healthy and GRMD dogs. C. QTcB (Bazett’s correction) overcorrected the QT value, leading to a positive correlation with HR in the healthy dogs’ dataset. D. QTcV (Van de Water’s correction) was the best correction formula in decorrelating QT from HR in our healthy dog cohort, consistently with other descriptions in the canine species. E. QTcF (Friedericia’s correction) in the healthy dogs’ dataset F. QTcM (Matsunaga’s correction) in the healthy dogs’ dataset

Figure S3 : Longitudinal ECG and HRV data including yearly follow-up of GRMD dogs after 24 months of age.

Figure S4: Serum cardiac biomarkers in the studied cohort

**A.** Serum Troponin I was increased to some very high values attesting to myocardial damage in GRMD dogs (logarithmic scale), though the difference relative to healthy dogs was only significant at the age of 4 months. **B.** NT-proBNP values were slightly increased in some GRMD dogs at 2 months of age, and then remained normal until 12 months when the blood NT-proBNP concentration progressively increased again. NT-proBNP values were significantly increased at the age of 24 months and reached very high values in some GRMD dogs.
